# Clinical and immunological evaluation of late diagnosed patients with HIV/AIDS infection in the Republic of Moldova

**DOI:** 10.1186/1471-2334-14-S7-P17

**Published:** 2014-10-15

**Authors:** Ina Bîstrițchi, Tiberiu Holban, Constantin Spînu

**Affiliations:** 1 Department of Infectious Diseases, Parasitology and Tropical Medicine, Nicolae Testemițanu State Medical and Pharmacy University, Chişinău, Republic of Moldova; 2 National Public Health Center, Republic of Moldova

## Background

HIV/AIDS infection is one of the priority issues of public health both globally as well as in Republic of Moldova. The negative impact of late presentation and detection is important for the health system, by increasing mortality and morbidity through requiring additional resources and for the community by transmission of HIV.

## Methods

We assessed 149 treatment-naïve patients with HIV/AIDS who initiated HAART in 2011-2012. The age of patients included in this study were between 18 and 77 years (36.3±0.8 years), out of which 60% were young people between 30 and 39 years. Late diagnosis was defined as the presence of AIDS related diseases and/or CD4 <350 cells/cmm.

## Results

The following modes of transmission were identified: heterosexual – 130 (87.3%) patients and IDUs (injecting drug use) – 19 (12.7%) patients. Out of 149 patients, 94 (63.1%) patients were detected late with CD4 counts <350 cells/cmm, out of which 56 (59.6%) patients were detected very late with CD4 counts <200 cells/cmm. HIV/AIDS infection was diagnosed during the period 1997-2011, and HAART was initiated in 2011. The period from detection of HIV infection to HAART initiation was less than 1 year to 1/3 of all patients – 47 (31.6%), 1 year – 21 (14.1%), 2 years – 15 (10.1%), 3 years – 20 (13.4%), 4 years – 15 (10.1%), 5 years – 10 (6.7%), 6 years – 5 (3.3%), 7 years – 6 (4%), 9 years – 2 (1.3%), 10 years – 1 (0.7%), 11 years – 3 (2%), 13 years – 3 (2%) and 14 years – 1 (0.7%). The average CD4 cell count at detection of HIV infection was 283.54±16.2 cells/cmm, and lower at initiation of HAART (220.5±11.3 cells/cmm), (p <0.01). At detection of HIV infection approximately 1/3 of patients (30.9%) had viral load >100000 copies/mL.

## Conclusion

About 2/3 (63.1%) of patients with HIV/AIDS are detected late, compared to 15-38% of the cases in the European Union, thus it is necessary to improve HIV testing strategies. The route of transmission of HIV infection was heterosexual – 87.3% and through injecting drug use – 12.7%. It was established that at the start of HAART the most common opportunistic infections were oropharyngeal candidiasis – 82 (55%) patients, tuberculosis – 34 (22.8%), wasting syndrome - 19 (13.1%), herpes zoster – 12 (8.3%), Kaposi's sarcoma – 4 (2.8%). At the initiation of HAART 110 (73.8%) were diagnosed with AIDS.

